# A Case of Impalement Brain Injury That Could Achieve Good Neurological Outcome by Introducing Early Sedation and Immobilization Strategy

**DOI:** 10.1155/2018/3025717

**Published:** 2018-04-01

**Authors:** Wataru Takayama, Kaho Yamasaki, Akira Endo, Yasuhiro Otomo

**Affiliations:** Trauma and Acute Critical Care Medical Center, Tokyo Medical and Dental University Hospital of Medicine, 1-5-45 Yushima, Bunkyo-ku, Tokyo, Japan

## Abstract

Impalement brain injury is rare, and the initial management of this condition is not well-established. We present a case of a well-managed brain injury caused by impalement with a metal bar. A 29-year-old man whose head had been impaled by a metal bar was transferred to our hospital. Upon arrival, he was agitated, with an unsteady gait and prominent odor of alcohol on his breath. He exhibited normal vital signs and neurological findings, except for his level of consciousness. To address the risk of secondary brain injury caused by movement of the foreign body, we immediately administered a sedative agent and muscle relaxant after the initial neurological evaluation. The imaging evaluation revealed the insertion of a metal bar into the right frontal lobe at a depth of >100 mm through the frontal bone; however, there was no apparent major vessel injury-related complication. Three hours after arrival at the hospital, a craniotomy was performed to remove the foreign body. The patient's postoperative course was uneventful, and he was discharged after rehabilitation without any neurological deficits. The strategy of immediate immobilization to prevent the secondary brain injury is important in the initial management of a patient who has survived an impalement brain injury and presented to an emergency department.

## 1. Introduction

Most patients with a penetrating brain injury (PBI) die before arriving at a hospital or emergency department, and survivors are at a high risk of morbidity from neurologic sequelae. However, some cases with good neurological outcomes have been reported [1, 2]. The management of impalement brain injury, an extremely rare type of PBI, depends on the case, and an initial management strategy has not been well-established. Here, we report a rare case of a well-managed impalement brain injury caused by the insertion of a hook-shaped metal bar (jacking device for automobiles) into the right frontal lobe through the frontal bone.

## 2. Case Report 

**Figure 1:**
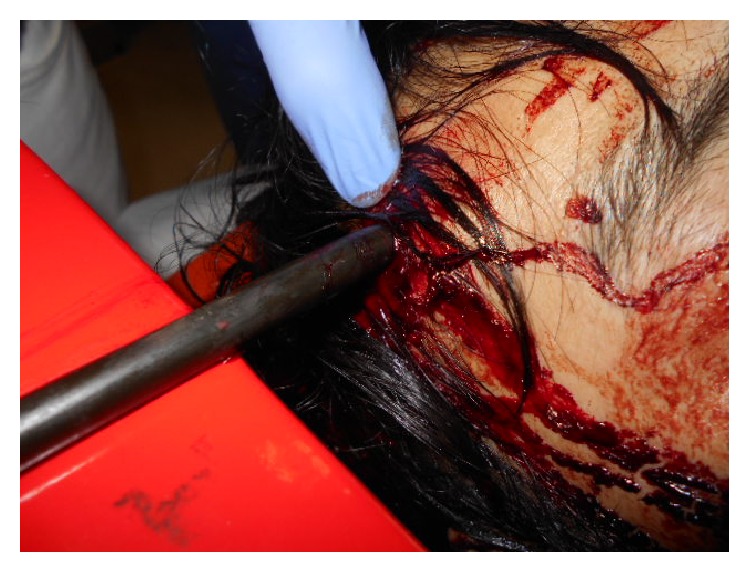
Photograph of a metal bar that had penetrated the right parietal region.

A 29-year-old man whose head was impaled with a metal bar was transferred to our hospital by ambulance (Figure 1). He was agitated, with an unsteady gait and a prominent odor of alcohol on his breath. His airway was patent and breathing was sufficient. Upon admission, his vital signs were as follows: pulse rate, 98/minute; blood pressure, 120/82 mmHg; respiratory rate, 20 breaths/minute; and body temperature, 36.8°C. He was hemodynamically stable with no apparent complicating injury. His Glasgow Coma Scale score was E2 V3 M5, and no neurological deficits were observed. His pupils were 4 mm/4 mm and reactive.

**Figure 2:**
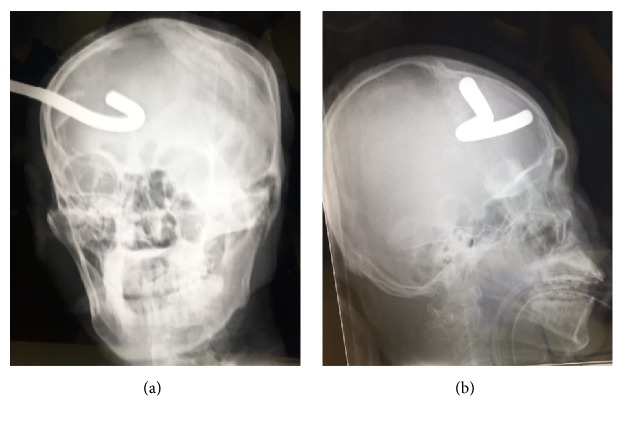
(a) Plain X-ray of the skull (A-P view) showing a hook-shaped metal bar on the right side. (b) Plain X-ray of the skull (R-L view) showing the metal bar in the frontal region.

**Figure 3:**
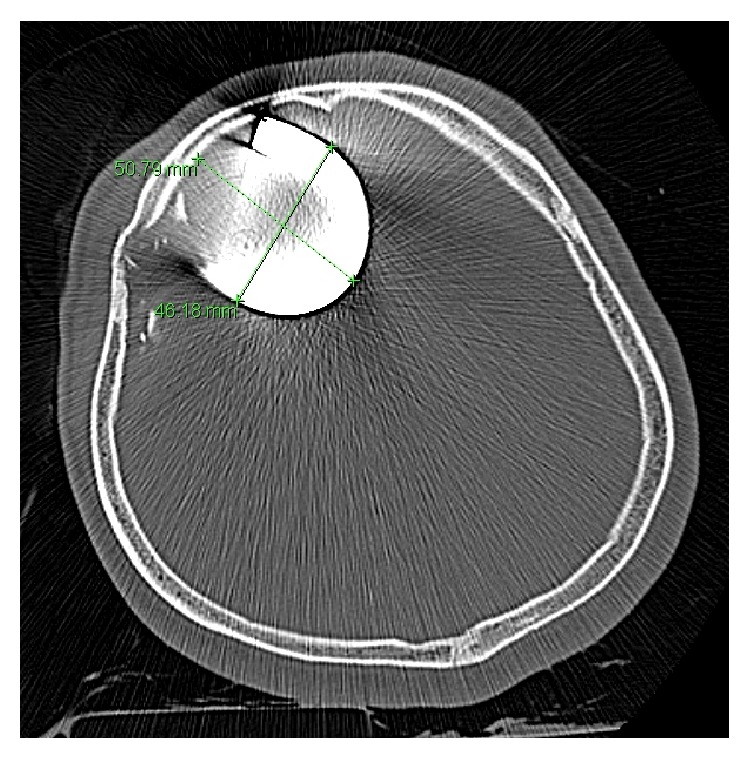
Axial computed tomography of the head (bone window), showing the intracranial penetration of a hook-shaped metal bar measuring 50.8 mm × 46.2 mm.

Given the risk of secondary brain injury caused by rotational movement of the foreign body with the skull as a fulcrum, we immediately administered a sedative agent and muscle relaxant after the initial neurological evaluation. Skull X-ray showed a 10 mm wide metal bar inserted into the right frontal region of the head at a depth of >100 mm (Figures 2(a) and 2(b)). Computed tomography (CT) was performed after the exposed part of the foreign body was cut using a hydraulic clamp, as it was too long to pass into the CT scanner gantry. CT of the head showed a hook-shaped metal bar measuring 51 mm × 46 mm in the right frontal lobe via the frontal bone (Figure 3). Cerebral CT angiography showed no apparent major vessel injury-related complication.

**Figure 4:**
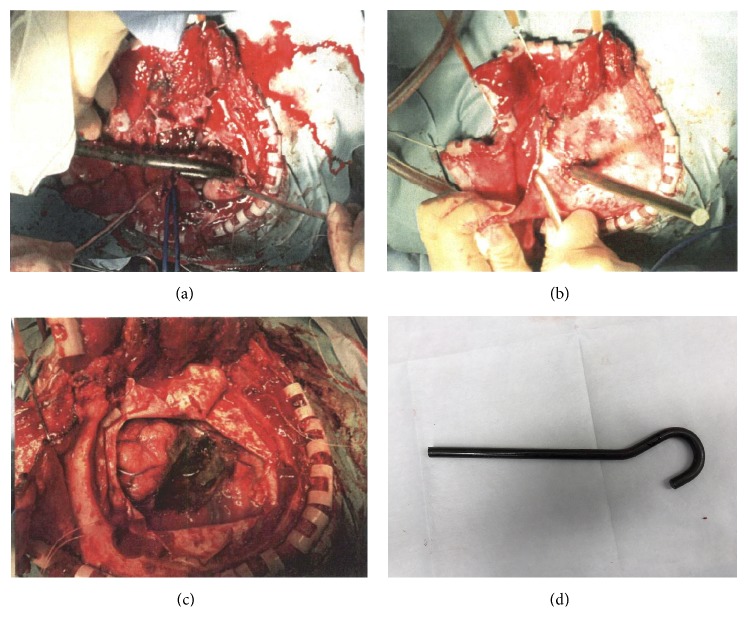
Intraoperative photographs. (a) Exposure of the metal bar after a semicircular incision. (b) The hook-shaped metal bar is shown to penetrate the frontal lobe without causing injuries to major arteries. (c) Neither bleeding nor hematoma was observed after removal. (d) The metal bar after surgical removal.

Three hours after hospital arrival, a craniotomy was performed to remove the foreign body. A-horse-shoe flap was created around the metal bar (Figure 4(a)), and the bone fixed under the foreign body was removed to prevent unnecessary movement. No major vessel injury or hematoma formation was observed intraoperatively, and the foreign body was removed under direct visualization (Figures 4(b), 4(c), and 4(d)). The patient's postoperative course was uneventful. He was extubated on postoperative day 1 and discharged after rehabilitation without any neurological deficits.

## 3. Discussion

We experienced a very rare case of impalement brain injury caused by a metal bar with a complicated shape, and successful patient management led to a good neurological outcome. PBI is among the most lethal forms of brain injury; 70–90% of affected patients with PBI die prior to arrival at a hospital, and 50% of survivors die during initial resuscitation [3, 4]. In cases of a stab or impalement injury, it is generally important to leave the foreign body in place and ensure that both the object and patient remain immobilized until the preparation for removal is complete [5]. However, cases involving a foreign body penetrating into the brain face an extremely high risk of secondary injury because the foreign body can easily rotate with the skull as a fulcrum [6], particularly if the patient is under the influence of alcohol or drugs. Therefore, for fortunate survivors who arrive at an emergency department, maximum effort should be made to prevent secondary injury to the brain caused by mobilization of the patient and/or foreign body. Accordingly, an initial management strategy involving the immediate immobilization of a patient with a penetrating foreign body in the brain is considered quite reasonable.

The prognosis of a patient with penetrating trauma generally depends on the site of injury and depth of penetration; in other words, the mortality and neurological outcome are essentially determined at the time of injury [4]. Although consequent vessel injury affects both mortality and morbidity, impalement injury is generally considered to be less frequently associated with vessel injury, compared to stab injury [5]; therefore, impalement injury (including brain injury) might have a better outcome than other types of penetrating injury. These principles and our experience suggest that a patient with an impalement brain injury who survives until arrival at an emergency department and has no severe neurological deficits could achieve a good neurological outcome if the adequate initial management strategy includes the following: (1) immediate immobilization of the patient and foreign body after neurological evaluation, (2) adequate evaluation of vessel injury, and (3) careful removal of the foreign body.

In conclusion, we encountered a rare and well-managed case of an impalement brain injury caused by a hook-shaped metal bar. Strategies to minimize secondary brain injury are important for the initial management of an impalement brain injury.
